# A Comparison of Entecavir and Lamivudine for the Prophylaxis of Hepatitis B Virus Reactivation in Solid Tumor Patients Undergoing Systemic Cytotoxic Chemotherapy

**DOI:** 10.1371/journal.pone.0131545

**Published:** 2015-06-29

**Authors:** Wen-Chi Chen, Jin-Shiung Cheng, Po-Hung Chiang, Feng-Woei Tsay, Hoi-Hung Chan, Hsueh-Wen Chang, Hsien-Chung Yu, Wei-Lun Tsai, Kwok-Hung Lai, Ping-I Hsu

**Affiliations:** 1 Division of Gastroenterology, Department of Medicine, Kaohsiung Veterans General Hospital, Kaohsiung, Taiwan; 2 School of Medicine, National Yang-Ming University, Taipei, Taiwan; 3 Department of Chemistry, College of Science, National Kaohsiung Normal University, Kaohsiung, Taiwan; 4 Department of Sport, Health & Leisure, Cheng Shiu University, Kaohsiung, Taiwan; 5 Department of Biological Sciences, National Sun Yat-Sen University, Kaohsiung, Taiwan; National Yang-Ming University, TAIWAN

## Abstract

**Background:**

Nucleos(t)ide analogues reduce the incidence of hepatitis B virus (HBV) reactivation in cancer patients undergoing systemic cytotoxic chemotherapy but the experience of solid tumors remains limited. Aims. The aim of this study was to compare the efficacy of entecavir and lamivudine in the prophylaxis of HBV reactivation in solid tumor patients undergoing systemic cytotoxic chemotherapy.

**Methods:**

HBsAg seropositive patients undergoing systemic cytotoxic chemotherapy for solid tumors with prophylactic entecavir and lamivudine between January 2006 and June 2013 were retrospectively investigated. The incidence of HBV reactivation and outcome of the patients were analyzed. The risk factors of HBV reactivation were examined.

**Results:**

A total of 213 patients (entecavir group, 70 patients; lamivudine group, 143 patients) were evaluated. Less incidence of HBV reactivation was noticed in entecavir group than in lamivudine group (0% vs. 7.0%, P = 0.02). No HBV reactivation was noticed in the patients with a baseline HBV DNA level < 2000 IU/mL. A baseline HBV DNA level ≥ 2000 IU/mL, HBeAg, and lamivudine were significantly associated with HBV reactivation. Subgroup analysis of the patients with a baseline HBV DNA level ≥ 2000 IU/mL found that lamivudine was significantly associated with HBV reactivation. Most of the reactivation events were properly managed by using tenofovir disoproxil fumarate. The incidence of hepatitis during chemotherapy and disruption of chemotherapy was similar between patients using entecavir and lamivudine with a baseline HBV DNA level ≥ or < 2000 IU/mL.

**Conclusions:**

A baseline HBV DNA level ≥ 2000 IU/mL, HBeAg, and lamivudine were the risk factors of HBV reactivation during systemic cytotoxic chemotherapy in solid tumor patients. Entecavir was superior to lamivudine in terms of less incidence of reactivation in the patients with a baseline HBV DNA level ≥ 2000 IU/mL. Both agents were equally efficacious in the patients with HBV DNA levels < 2000 IU/mL.

## Introduction

Hepatitis B virus (HBV) infection is a global public health problem. It is estimated that there are 350 to 400 million HBV carriers in the world, of whom roughly one million die from HBV-related liver disease annually [1]. Cancer patients seropositive for HBsAg are at a risk of HBV reactivation during systemic cytotoxic chemotherapy (SCC) or after withdrawal of cytotoxic agents because of a rebound of immune response [2]. Reactivation of HBV usually occurs after the first 2–3 cycles of SCC and is characterized by rapid raise of serum HBV DNA levels followed by elevation of aminotransferase levels and even liver decompensation [3]. The incidence of HBV reactivation approximately ranged from 20% to 70% among HBV carriers receiving SCC and the mortality rate ranged between 5% and 40% [3–5]. This is particularly a major problem for cancer patients who need SCC in HBV endemic area.

Several risk factors associated with HBV reactivation during SCC including male gender, younger age, HBeAg seropositivity, steroid-containing or anthracycline-containing SCC, lymphoma, high pre-SCC alanine aminotransferase (ALT) level, high pre-SCC HBV DNA level, and high pre-SCC intrahepatic covalently closed circular DNA level were reported [5]. Recently, biological agents such as rituximab were also found to increase the risk of HBV reactivation in 2% to 25% of hematological patients [6]. Of these factors, a high HBV DNA level at baseline is the best predictor of HBV reactivation and is associated with a higher incidence of severe hepatitis during SCC and poor survival [7]. This suggests that HBV DNA level should be specifically concerned in the management of HBV-infected patients during SCC.

In contrast to hematological tumors, the incidence of HBV reactivation associated with SCC ranged from 20% to 36% in HBsAg seropositive patients with solid tumors including hepatocellular carcinoma, breast cancer, lung cancer, and nasopharyngeal cancer [8–11]. Anthracycline-containing SCC was found to increase the risk of HBV reactivation in HBV-infected patients with solid tumors [12]. However, the association of HBV loads with HBV reactivation in solid tumor patients was rarely investigated.

Prophylactic antivirals are superior to deferred preemptive antivirals to prevent HBV reactivation during SCC [13]. Among the available nucleos(t)ides, lamivudine (LAM) gains the most experience in reactivation prophylaxis during SCC [14]. Nevertheless, LAM is a weak nucleoside with a mutation rate of 23% after 1 year and up to 65% after 5 years in nonimmunocompromised patients [15] and the rate of resistance would be anticipated to be even higher in patients undergoing SCC. Emergence of HBV mutants resistant to LAM is associated with rapid clinical deterioration in immunosuppressed patients [16] and this should be significantly considered in the prophylaxis of HBV reactivation during SCC.

Third-generation nucleos(t)ide analogue such as ETV is superior to LAM in the suppression of HBV replication with an extremely low mutation rate in both HBeAg-positive and HBeAg-negative patients [17,18]. In the case of lymphoma and hematological malignancies, ETV was found to be associated with a significantly lower rate of HBV reactivation than LAM [19–22]. Up to now, no study compared the prophylactic efficacy of ETV and LAM in solid tumor patients undergoing SCC. This study aimed at comparing the efficacy of ETV and LAM in the prophylaxis of HBV reactivation in these patients.

## Materials and Methods

In Taiwan, nucleos(t)ides were reimbursed for HBV reactivation prophylaxis during SCC in HBsAg seropositive cancer patients within a timeframe between 1 week prior to the start of SCC and 6 months after the completion of SCC as the suggestion by the APASL guideline [23]. Long-term antivirals were reimbursed in the cirrhotic patients with HBV DNA levels > 2000 IU/L. Clinical assessment, liver biochemical tests, and serological hepatitis B markers were monitored at 2 to 3-month intervals during and after cessation of antiviral treamtent in our hospital. HBV DNA was monitored at 3 to 6-months intervals or suspicion of reactivation during antiviral treamtent and suspicion of reactivation after cessation of treamtent. We retrospectively reviewed the medical records of the cancer patients undergoing SCC between January 2006 and June 2013 and the data was retrieved for analysis.

### Patients

Patients were considered to be included into this study if they met the following criteria: (1) aged equal to or more than 20 years old; (2) solid tumor patients undergoing SCC; (3) seropositive for HBsAg at the entry of this study; and (4) using ETV 0.5 mg once daily or LAM 100 mg once daily for the prophylaxis of HBV reactivation. The exclusion criteria were: (1) seropositive for hepatitis C virus, hepatitis D virus, and human immunodeficiency virus; (2) alcoholism; (3) a baseline serum ALT > 80 U/L or a serum total bilirubin > 2.0 mg/dL before SCC; (4) a baseline serum creatinine > 2.0 mg/dL; (5) discontinuing ETV or LAM during SCC (6) a history of exposure to any nucleos(t)ide.

### Methods

The demographic data of the patients including age, gender, cancer types, duration of SCC, serum ALT level, total bilirubin level, status of HBeAg, HBV DNA level at the initial point, during SCC, and within 1 year after stopping ETV or LAM were retrieved from the medical records. We evaluated the incidence of reactivation, acute hepatitis, and liver decompensation due to HBV during the administration of ETV and LAM and after stopping ETV and LAM. Subgroup analysis of the patients with a baseline serum HBV DNA level equal to/higher than or lower than 2000 IU/mL was also conducted.

HBsAg titers were measured using ARCHITECT HBsAg assay (Abbott Diagnostics, Santa Clara, CA, USA) and specimens with concentration values ≥ 0.05 IU/mL were considered reactive. HBeAg titers were measured using ARCHITECT HBeAg assay (Abbott Diagnostics, Santa Clara, CA, USA) and specimens with S/CO values ≥ 1.000 were considered reactive. HBV DNA titers were measured with COBAS TaqMan HBV test (Roche Molecular Systems, Inc., South Branchburg, NJ, USA) with a lower detection limit of 6 IU/mL. LAM mutant was determined by direct sequencing of the PCR amplification products. To detect the mutations, the upstream primer 5′- TCACTCACCAACCTCTTGTC-3′ and the downstream primer 5′- AAGTGTTTGCTGACGCAACC-3′ were used for PCR amplification, and the primer 5′- GTAATTCCCATCCC- 3′ was used for sequencing. The PCR amplifications were performed in a PCR mechanism under the following conditions: after an initial denaturation for 5 min at 94° C, samples were subjected to 35 cycles of amplification (94° C 30 s, 55° C 30 s, 72° C 1 min), followed by a final extension of 5 min at 72° C. The purification of PCR products was performed by QIAquick PCR purification kit according to manufacture’s instructions (QIAGEN, Valencia, CA, USA). Sequence analysis of the PCR products was performed by BigDye Direct Cycle Sequencing Kit (Applied Biosystems, Thermo Fisher Scientific Inc., Waltham, MA, USA) in a ABI 3730XL according to manufacture’s instructions. Sequence analysis software was used to analyze the results.

### Definitions

Prophylactic nucleos(t)ides were defined as antiviral prophylaxis given 1 week prior to the start of chemotherapy and 6 months after the completion of SCC. A high HBV DNA level was defined as a HBV DNA level ≥ 2000 IU/mL [24]. A low HBV DNA level was defined as a HBV DNA level < 2000 IU/mL. HBV reactivation was defined as an increase in HBV DNA levels of 10-fold or more compared with the previous nadir levels [25]. Delayed HBV reactivation was defined as HBV reactivation after discontinuing prophylactic antivirals [26]. The severity of hepatitis was defined as “mild” (the increase in ALT < 80 U/L), “moderate” (ALT between 80 U/L and 200 U/L), and “severe” (ALT > 200 U/L). Disruption of SCC was defined as delay of SCC more than 8 days in between cycles of SCC or premature termination of SCC [27].

### Study end points

The primary end points of this study were reactivation of HBV during and after the use of ETV and LAM and hepatitis or liver decompensation associated with HBV. The secondary end points were disruption of SCC and mortality.

### Statistical analysis

Demographic data and the events of primary and secondary end points were compared between both groups. Categorical data were compared using chi-square or Fisher’s exact test as appropriate. Continuous variables with normal distributions were compared using independent Student’s t test. Continuous variables without normal distributions were compared using Mann-Whitney U test. Kaplan-Meier estimation was conducted to examine the time to occurrence of HBV reactivation in the patients with a baseline HBV DNA level ≥ 2000 IU/mL during the use of nucleos(t)ides and after the discontinuation of nucleos(t)ides. A log rank test was used to examine the variation of HBV reactivation between the patients using ETV and LAM. Univariate and multi-variate analysis using logistic regression model with Firth's bias correction, chi-square test, Fisher’s exact test, or Mantel-Haenszel test were used to examine the risk factor of HBV reactivation when appropriate. Significance was defined as P < 0.05 for all two-tailed tests. All the analyses were conducted by using SAS software (version 9.3, SAS Institute Inc., Cary, NC, USA).

### Ethics Statement

This cohort study was approved by the Institutional Review Board of Kaohsiung Veterans General Hospital (VGHKS13-CT6-12) and has been conducted according to the principles expressed in the Declaration of Helsinki. No consent was given because the data were analyzed anonymously.

## Results

### Characteristics of patients

Between January 2006 and June 2013, there were 298 HBsAg seropositive solid tumor patients undergoing SCC in this hospital. Eighty-five patients were excluded: seropositive for hepatitis C virus, 16 patients; alcoholism, 8 patients; a baseline serum ALT > 80 U/L; 23 patients; a baseline serum total bilirubin > 2.0 mg/dL, 4 patients; discontinuing ETV or LAM during SCC, 2 patients; a history of exposure to any nucleos(t)ide, 32 patients. A total of 213 eligible patients with prophylactic ETV or LAM therapy were analyzed. The selection of prophylactic antivirals depended largely on the preference of the caring physicians. There were 70 patients in ETV group and 143 patients in LAM group. The demographic data of the patients including age, gender, serum ALT levels, serum HBV DNA levels before SCC, status of HBeAg, and duration of antiviral prophylaxis were similar between ETV group and LAM group while patients in ETV group had a higher proportion of hepatoma and liver cirrhosis (Table 1).

**Table 1:** Demographic data of solid tumor patients undergoing systemic cytotoxic chemotherapy and using prophylactic entecavir or lamivudine.

Variables	Entecavir group (n = 70)	Lamivudine group (n = 143)	P value
Age (year)	56.5±10.3	56.0±10.3	0.8
Male gender	42 (60.0%)	76 (53.1%)	0.3
Tumor types			
Hepatoma	25 (35.7%)	15 (10.5%)	< 0.001
Breast cancers	9 (12.9%)	26 (18.2%)	0.3
Lung cancer	8 (11.4%)	24 (16.8%)	0.3
Gastrointestinal cancers	11 (15.7%)	21 (14.6%)	0.8
Other cancers^*a*^	17 (24.3%)	57 (39.9%)	0.03
Anthracycline-containing SCC^*b*^	18 (25.7%)	29 (20.3%)	0.4
Cirrhosis	23 (32.8%)	14 (9.8%)	< 0.001
ALT^*c*^ (U/L)	37.4±20.4	33.6±21.8	0.2
INR^*d*^	1.1±0.1	1.0±0.1	0.06
HBeAg	5 (7.1%)	11 (7.7%)	0.9
HBV DNA level ≥ 2000 IU/mL	34 (48.6%)	62 (43.3%)	0.5
Duration of prophylaxis (mon.)	9.1±3.8	8.4±4.6	0.3
Follow-up (mon.)	15.4±7.4	14.4±6.0	0.3

^*a*^Entecavir group included 6 head and neck cancers, 4 gynecologic cancers, and 7 genitourinary cancers. Lamivudine group included 31 head and neck cancers, 15 gynecologic cancers, and 11 genitourinary cancers.

^*b*^SCC: systemic cytotoxic chemotherapy

^*c*^ALT: alanine aminotransferase.

^*d*^INR: international normalized ratio.

We further divided the patients into high baseline HBV DNA level (≥ 2000 IU/mL) patients and low baseline HBV DNA level (< 2000 IU/mL) patients. The demographic data of the patients with high baseline HBV DNA levels were shown in S1 Table. Patients in ETV group and LAM group with a high HBV DNA level had similar age, gender, serum ALT levels, serum HBV DNA levels before SCC, status of HBeAg, and duration of antiviral prophylaxis while patients in ETV group had a higher proportion of hepatoma and liver cirrhosis than patients in LAM group (58.8% vs. 17.7%; P < 0.001 and 52.9% vs. 17.7%; P < 0.001, respectively).

The demographic data of the patients with a baseline HBV DNA level < 2000 IU/mL were shown in S2 Table. Patients in ETV group and LAM group had similar age, gender, serum ALT levels, status of HBeAg, baseline HBV DNA levels, proportion of cirrhosis, and duration of antiviral prophylaxis.

### Characteristics and outcome of antivirals prophylaxis

**Fig 1:**
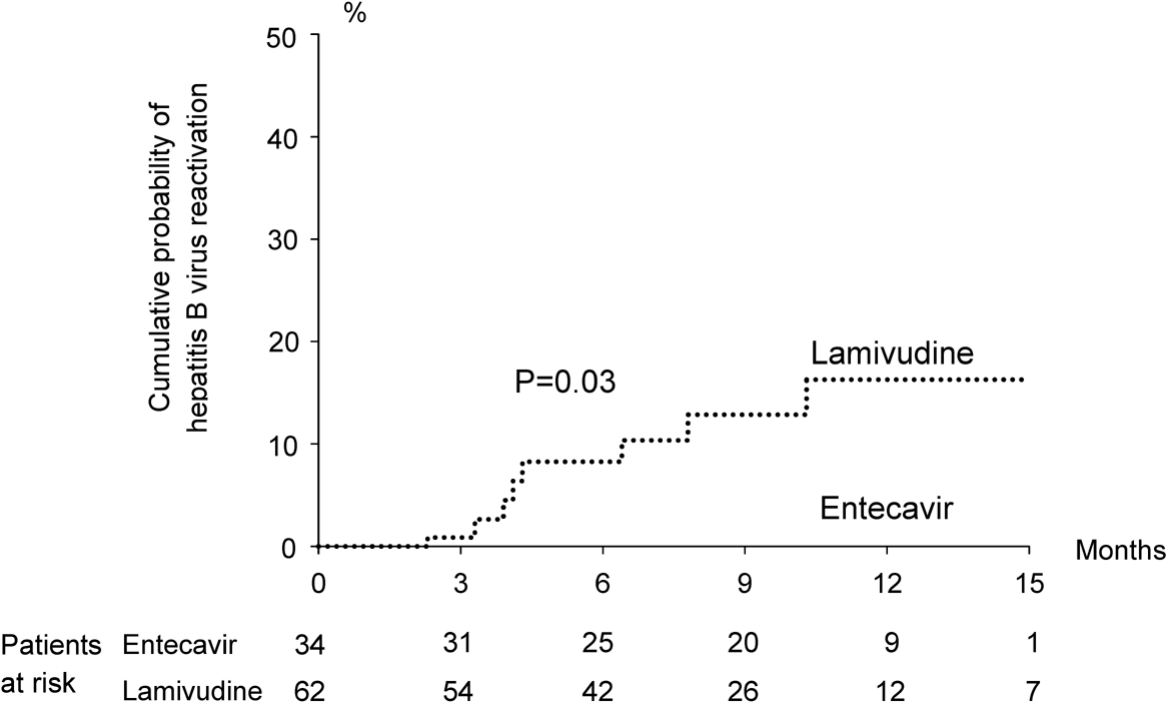
Hepatitis B reactivation during systemic cytotoxic chemotherapy in solid tumor patients with a baseline HBV DNA level equal to or more than 2000 IU/mL and using prophylactic entecavir and lamivudine.

Significantly less incidence of HBV reactivation was noticed in entecavir group (0 of 70 patients, 0%) than in lamivudine group (10 of 143 patients, 7.0%) (P = 0.02). Of the patients with a baseline HBV DNA level ≥ 2000 IU/mL, no patient (0%) in ETV group had HBV reactivation while HBV reactivation was noticed in 10 patients (15.9%) in LAM group (P = 0.01). The cumulative incidence of HBV reactivation in ETV group and LAM group patients was shown in Fig 1. Hepatitis was noticed in 2 patients (5.9%) in ETV group (mild, 2 patients) and 8 patients (12.9%) in LAM group (mild, 3 patients; moderate, 3 patients; severe, 2 patients) (P = 0.5). Disruption of SCC due to hepatitis was noticed in 1 patient (2.9%) in ETV group and 6 patients (9.7%) in LAM group (P = 0.4). Of the patients with a baseline HBV DNA level < 2000 IU/mL, no patients had HBV reactivation during SCC in both ETV and LAM group. Hepatitis was noticed in 2 patients (5.6%) in ETV group (mild, 1 patient; severe, 1 patient) and 15 patients (18.5%) in LAM group (mild, 9 patients; moderate, 4 patients; severe, 2 patients) (P = 0.2). Disruption of SCC due to hepatitis was noticed in 1 patient (2.8%) in ETV group and 9 patients (11.1%) in LAM group (P = 0.2).

Age, gender, prophylactic nucleos(t)ide analogues, tumor types, ALT level, HBeAg status, and HBV DNA level were examined to estimate the risk factors of HBV reactivation in the entire cohort. During univariate analysis, a baseline HBV DNA ≥ 2000 IU/mL (OR: 28.5, 95% CI: 1.7–493.4, P < 0.0001; logistic regression model), HBeAg (OR: 10.6, 95% CI: 2.6–43.5, P = 0.0009; logistic regression model), and LAM (P = 0.03; chi-square test) were found to be significantly associated with HBV reactivation. With multi-variate analysis, the maximum likelihood estimate for a baseline HBV DNA ≥ 2000 IU/mL did not exist due to complete separation, even with Firth's bias correction. In the patients with a baseline HBV DNA ≥ 2000 IU/mL, univariate analysis found that LAM (P = 0.01; chi-square test) and HBeAg (OR: 4.6, 95% CI: 1.1–18.9, P = 0.04; logistic regression model) were associated with HBV reactivation. Multi-variate analysis was performed to estimate the significance but complete separation was encountered. Use of LAM controlled for HBeAg by Mantel-Haenszel test found that LAM was significantly associated with HBV reactivation (P = 0.02).

Of the patients with HBV reactivation during SCC, LAM resistant mutants were noticed in 4 patients. Rescue strategies were adopted according to the clinical judge of the caring physicians. The median follow-up time of salvage antiviral treatment was 15.9 (10.3–23.1) months. Salvage tenofovir disoproxil fumarate (TDF) 300 mg daily was used in 8 patients. The median Log_10_[HBV DNA] were 5.1 (3.9–9.2) IU/mL at reactivation and 3.1 (undetectable to 5.4) IU/mL at 3rd month. HBV DNA levels were undetectable in 7 patients and 1 patient had a Log_10_[HBV DNA] of 3.6 IU/mL at 6th month. HBV DNA levels were undetectable in 8 patients at 9th months. Entecavir 1 mg daily was used in 1 patient and the Log_10_[HBV DNA] was 5.9 IU/mL at reactivation, 3.3 IU/mL at 3rd month, and undectable at 6th month. One patient was observed for 14.1 months and the Log_10_[HBV DNA] was 3.9 IU/mL at reactivation, 3.2 IU/mL at 3rd month, 3.1 IU/mL at 6th month and 3.3 IU/mL at 12th month. The characteristics and outcome of the LAM prophylaxis failure patients were shown in Table 2.

**Table 2:** Occurrence and outcome of hepatitis B reactivation during systemic cytotoxic chemotherapy in HBsAg-positive patients.

Patient No.	Tumor type	Start of SCC to reactivation (months)	Log_10_[HBV DNA level] (IU/mL)			Lamivudine resistance mutant	ALT^*b*^ at reactivation (U/L)	Total bilirubin at reactivation (mg/dL)	Management	Outcome
			Pre-SCC^*a*^	Nadir	Reactivation					
1	Breast	6.4	6.4	4.7	9.2	M204I	249	0.5	Tenofovir	Mortality
2	Liver	4.3	6.1	4.1	5.8	NI	49	0.5	Tenofovir	Survive
3	Esophagus	3.9	5.0	3.2	5.1	NI	25	0.4	Tenofovir	Survive
4	Lung	4.1	7.2	3.5	5.9	NI	67	0.8	Entecavir	Survive
5	Ovary	2.3	7.4	3.9	5.6	NI	210	1.2	Tenofovir	Survive
6	Liver	10.3	4.0	1.2	3.9	NI	43	0.6	Tenofovir	Survive
7	Breast	7.8	6.6	2.8	4.1	M204I	23	0.4	Tenofovir	Survive
8	Breast	3.3	7.4	3.5	4.8	L180M	42	0.3	Tenofovir	Survive
9	Liver	26.0	5.1	1.2	5.1	M204V	33	0.8	Tenofovir	Survive
10	Ovary	24.7	3.7	<0.8	4.0	NI	29	0.8	Observation	Survive

^*a*^SCC: cytotoxic chemotherapy.

^*b*^ALT: alanine aminotransferase.

Of the patients with high baseline HBV DNA levels, 14 of 34 patients (41.1%) using ETV died during SCC and 25 of 62 patients (40.3%) using LAM died (P = 0.9). Of the patients with a low baseline HBV DNA level, 12 of 36 patients (33.3%) using ETV died during SCC and 36 of 81 patients (44.4%) using LAM died (P = 0.3). No patient in this cohort died of liver failure associated with HBV reactivation.

**Fig 2:**
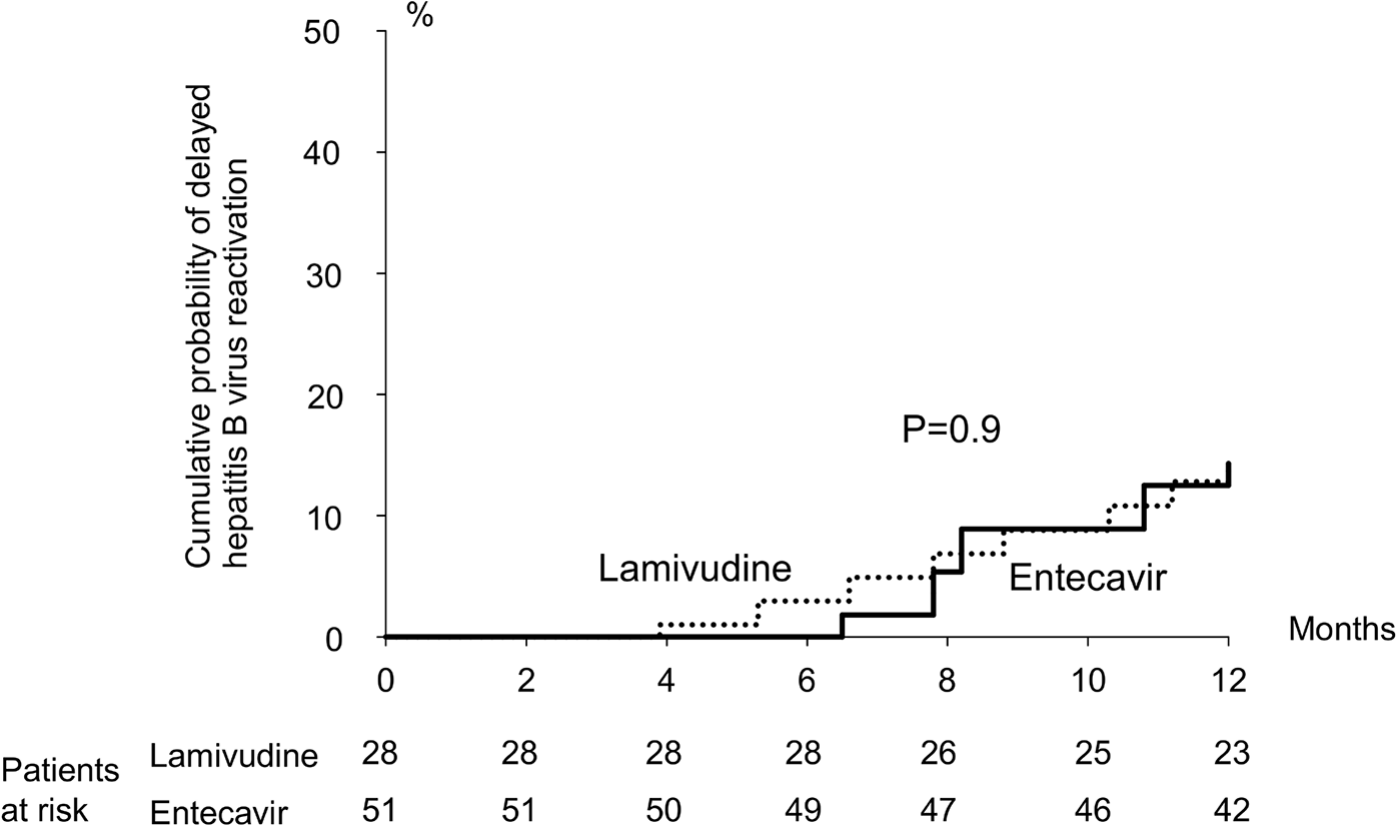
Delayed hepatitis B virus reactivation within 1 year after discontinuation of prophylactic entecavir and lamivudine in solid tumor patients undergoing systemic cytotoxic chemotherapy.

Twenty cirrhotic patients with high baseline HBV DNA levels were on long-term antivirals after SCC (ETV group, 12 patients; LAM group, 8 patients). Ten LAM group patients with HBV reactivation were also on long-term antivirals. Seventeen patients lost to follow-up after SCC (ETV group, 4 patients; LAM group, 13 patients). Totally 77 patients met the criteria of APASL guideline. Among the 79 patients completed SCC and discontinued antivirals, delayed HBV reactivation was noticed in 4 of 28 patients (14.3%; high baseline HBV DNA level, 3 patients; low baseline HBV DNA level, 1 patient) using prophylactic ETV and 7 of 51 patients (13.7%; high baseline HBV DNA level, 5 patients; low baseline HBV DNA level, 2 patients) using prophylactic LAM (Fig 2). All the patients with delayed HBV reactivation after withdrawal of ETV resumed ETV and delayed HBV reactivation was controlled. Of the patients with delayed HBV reactivation after withdrawal of LAM, TDF was started in 5 patients and 1 patient was observed without any antiviral treatment. All the patients recovered from delayed HBV reactivation soon.

## Discussion

The current study compared the efficacy of ETV and LAM in the prophylaxis of HBV reactivation during SCC in HBsAg seropositive patients with solid tumors using a strategy suggested by APASL guideline. A baseline HBV DNA level ≥ 2000 IU/mL, HBeAg, and LAM were found to be the risk factors of HBV reactivation. Subgroup analysis of the patients with a baseline HBV DNA level ≥ 2000 IU/mL found that LAM was significantly associated with HBV reactivation. Most of the HBV reactivation events were successfully managed after early administration of salvage TDF. On the other hand, no HBV reactivation was found in low baseline HBV DNA level patients using prophylactic ETV and LAM.

ETV and LAM were compared for the prophylaxis of HBV reactivation in lymphoma patients undergoing SCC [19–22]. A retrospective study by Li et al. found that patients using ETV had a significantly lower incidence of hepatitis, HBV reactivation, and disruption of chemotherapy compared with patients using LAM in lymphoma patients seropositive for HBsAg [21]. Chen et al. reported that ETV and LAM were both efficacious in the prophylaxis of HBV reactivation in HBcAg seropositive hematological disorder patents and ETV was preferred in patients with detectable baseline HBV DNA [20]. The only available prospective randomized controlled trial showed that ETV was superior to LAM with a significantly lower rate of HBV reactivation and disruption of SCC due to hepatitis B and the HBV reactivation in LAM patients was presumably due to drug resistance [19]. Nevertheless, whether the experience of lymphoma patients could apply to solid tumor patients was unknown.

High viral load at baseline is the most important risk factor for HBV reactivation associated with SCC [28]. Although LAM is the most common agent used in the prophylaxis of HBV reactivation during SCC, patients with a baseline HBV DNA titer >2,000 IU/mL were at a high risk of LAM prophylaxis failure [29]. For the patients with a high HBV DNA level and in whom a prolonged SCC is expected, a high genetic barrier nucleos(t)ide analogue is usually suggested [30]. We found that ETV was superior to LAM in the prophylaxis of HBV reactivation during SCC in solid tumor patients with a baseline HBV DNA level ≥ 2000 IU/mL. In our patients using prophylactic LAM, the incidence of HBV reactivation was up to 15.9%, which was similar to a previous study [29]. Our findings supported the recommendation of using ETV for the prophylaxis of HBV reactivation during SCC in solid tumor patients with a high baseline HBV DNA level.

The potential link of LAM mutants with the development of HBV reactivation in immunocompromised patients is still unknown [5] and the experience in the management of LAM mutants is limited. The emergence of LAM-resistant mutants is generally regarded as the main cause of HBV reactivation in patients undergoing SCC. However, not all HBV reactivation is attributable to LAM resistant mutants in LAM prophylaxis failure patients. LAM resistant mutants were found in 7 of 15 patients with HBV reactivation which occurred at a median of 10.9 months (range, 0.4 to 23.5 months) [29]. Another study showed that YMDD mutation was found in 7 of 12 patients with HBV reactivation [22]. HBV reactivation was noticed in 10 of our LAM patients with a baseline HBV DNA level ≥ 2000 IU/mL and LAM resistant mutants were found in 4 of these patients. It was possible that the immunosuppressive effect of SCC led to early rise of HBV DNA levels and absence of LAM resistant mutants. Besides, the intrinsic property of LAM might limit its ability to inhibit virus replication in some immunocompromised patients. For the patients with YMDD mutants, stopping LAM was associated with a risk of liver failure unsalvageable by other antivirals due to relapse of wild type virus [31]. Adefovir add-on treatment in patients with LAM-resistant chronic hepatitis B suppresses HBV replication more effectively than ETV 1.0 mg [32]. However, the probability of achieving undetectable HBV DNA levels by adding adefovir is low in LAM-resistant patients with HBV DNA > 10^6^–10^8^ copies/mL [33]. On the other hand, TDF induces a potent and long-lasting antiviral effect after failure of LAM and LAM-adefovir combination therapy [33].

Solid tumor patients with HBV DNA levels < 2000 IU/mL are usually considered as at a relative low risk of HBV reactivation during SCC and a less potent antiviral agent such as LAM is proposed to be adequate for the patients in whom a short duration of SCC is scheduled [6]. However, there was limited data about the use of prophylactic nucleos(t)ides in the patients with a low HBV DNA load. In our study, LAM and ETV were both highly efficacious in the prophylaxis of HBV reactivation in solid tumor patients with a low HBV DNA level and this finding supported the use of prophylactic LAM in these patients. In fact, it would be very difficult to compare the efficacy of both agents in a prospective manner in consideration of the low reactivation rate.

Up to 13% of the patients undergoing SCC had hepatitis flare after withdrawal of prophylactic LAM [5]. A prechemotherapy HBV DNA of > 10^4^ copies/mL was found to be the most important risk factor for HBV reactivation after withdrawal of preemptive LAM [34]. Prolonged LAM therapy until restoration of host immune control of HBV was suggested in patients with high prechemotherapy HBV DNA levels [28]. In our patients following the prophylactic strategy suggested by APASL guideline, delayed HBV reactivation was still noticed in 14.3% of the patients using ETV and 13.7% of the patients using LAM, respectively. Actually, 1-year HBV relapse rate of 45% was observed in patients off long-term ETV therapy [35]. To decrease the incidence of delayed HBV reactivation, it is recommended that patients with a baseline HBV DNA of > 2000 IU/mL should continue antivirals until treatment endpoints as in immunocompetent patients [30].

Some limitation did exist in this study. First, it was a retrospective cohort study and the results should be interpreted cautiously because of potential selection bias resulting from physician preference for drug assignment. We also could not exclude that the relative ineffectiveness of LAM compared to ETV and the absence of LAM resistance in 6 of 10 HBV reactivation patients could be due to the LAM-treated patients not closely adhering to their treatment regime. Second, the characteristics were different between ETV group and LAM group. More patients in ETV group had hepatoma and liver cirrhosis while more patients in LAM group had head and neck, gynecologic, and genitourinary cancers. However, no HBV reactivation was noticed in ETV group although patients with hepatoma and liver cirrhosis were both at a high risk of HBV reactivation associated with SCC [36]. Third, the HBV DNA testing was not reimbursed after discontinuation of prophylactic antivirals in Taiwan so the incidence of delayed HBV reactivation after discontinuation of antivirals could be underestimated. Finally, most of the enrolled patients were seronegative for HBeAg (ETV group, 92.9%; LAM group, 92.3%) and the results should apply mainly to HBeAg-negative patients with chronic HBV infection.

## Conclusions

In conclusion, this study found that a baseline HBV DNA level ≥ 2000 IU/mL, HBeAg, and LAM were the risk factors of HBV reactvation during SCC in solid tumor patients. ETV was superior to LAM in patients with a baseline HBV DNA level ≥ 2000 IU/mL in terms of less incidence of HBV reactivation. Most the reactivation events were properly managed by rescue TDF. We also found that ETV and LAM were both highly efficacious in reactivation prophylaxis in solid tumor patients with a baseline HBV DNA level < 2000 IU/mL. More future studies are required to investigate and avoid the occurrence of delayed HBV reactivation after discontinuing nucleos(t)ide analogues in patients undergoing SCC.

## Supporting Information

S1 Data Underlying participant-level data are provided in a supporting information file (data.xls). (XLS)

S1 Table Demographic data of solid tumor patients with baseline HBV DNA levels equal to or more than 2000 IU/mL undergoing systemic cytotoxic chemotherapy. (DOC)

S2 Table Demographic data of solid tumor patients with baseline HBV DNA levels less than 2000 IU/mL undergoing systemic cytotoxic chemotherapy. (DOC)
