# Supplementary material for: A Comparison of Entecavir and Lamivudine for the Prophylaxis of Hepatitis B Virus Reactivation in Solid Tumor Patients Undergoing Systemic Cytotoxic Chemotherapy
Source: PLoS One. 2015 Jun 29;10(6):e0131545. doi: 10.1371/journal.pone.0131545 (PMC4488285; doi:10.1371/journal.pone.0131545)
Supplement: S1 Table — (DOC) [file pone.0131545.s002.doc]

S1 Table. Demographic data of solid tumor patients with baseline HBV DNA levels equal to or more than 2000 IU/mL undergoing systemic cytotoxic chemotherapy.

| Variables | Entecavir group (n=34) | Lamivudine group (n=62) | P value |
| --- | --- | --- | --- |
| Age (year) | 56.2±9.8 | 56.0±9.6 | 0.9 |
| Male gender | 23 (67.6%) | 35 (55.6%) | 0.2 |
| Cancer types |  |  |  |
| Hepatoma | 20 (58.8%) | 11 (17.7%) | < 0.001 |
| Breast cancer | 4 (11.8%) | 14 (22.6%) | 0.3 |
| Lung cancer | 2 (5.8%) | 5 (8.1%) | 1.0 |
| Gastrointestinal cancers | 3 (8.8%) | 9 (14.5%) | 0.5 |
| Other cancers*^a^* | 5 (14.8%) | 23 (37.1%) | 0.02 |
| Anthracycline-containing SCC*^b^* | 10 (29.4%) | 17 (27.4%) | 0.8 |
| Cirrhosis | 18 (52.9%) | 11 (17.7%) | < 0.001 |
| ALT*^c^* (U/L) | 42.5±21.5 | 38.8±23.8 | 0.5 |
| INR*^d^* | 1.1±0.1 | 1.0±0.1 | 0.06 |
| HBeAg | 4 (11.8%) | 11 (17.7%) | 0.6 |
| Log_10_[HBV DNA level (IU/mL)] | 5.6±1.4 | 5.0±1.3 | 0.1 |
| Duration of prophylaxis (mon.) | 9.3±4.2 | 8.5±5.7 | 0.5 |
| Follow-up (mon.) | 15.9±8.1 | 14.9±6.9 | 0.5 |

*^a^*Entecavir group included 1 head and neck cancer and 4 genitourinary cancers. Lamivudine group included 13 head and neck cancers, 5 gynecologic cancers, and 5 genitourinary cancers.

*^b^*SCC: systemic cytotoxic chemotherapy.

*^c^*ALT: alanine aminotransferase.

*^d^*INR: international normalized ratio.
