# Supplementary material for: A Comparison of Entecavir and Lamivudine for the Prophylaxis of Hepatitis B Virus Reactivation in Solid Tumor Patients Undergoing Systemic Cytotoxic Chemotherapy
Source: PLoS One. 2015 Jun 29;10(6):e0131545. doi: 10.1371/journal.pone.0131545 (PMC4488285; doi:10.1371/journal.pone.0131545)
Supplement: S2 Table — (DOC) [file pone.0131545.s003.doc]

S2 Table. Demographic data of solid tumor patients with baseline HBV DNA levels less than 2000 IU/mL undergoing systemic cytotoxic chemotherapy.

| Variables | Entecavir group (n=36) | Lamivudine group (n=81) | P value |
| --- | --- | --- | --- |
| Age (year) | 56.7±10.8 | 56.0±10.9 | 0.7 |
| Male gender | 19 (52.8%) | 41 (50.6%) | 0.9 |
| Cancer types |  |  |  |
| Hepatoma | 5 (13.9%) | 4 (4.9%) | 0.1 |
| Breast cancer | 5 (13.9%) | 12 (14.8%) | 1.0 |
| Lung cancer | 6 (16.7%) | 19 (23.4%) | 0.5 |
| Gastrointestinal cancers | 8 (22.2%) | 12 (14.9%) | 0.3 |
| Other cancers*^a^* | 12 (33.3%) | 34 (42.0%) | 0.4 |
| Anthracycline-containing SCC*^b^* | 10 (27.8%) | 21 (25.9%) | 0.9 |
| Cirrhosis | 5 (13.9%) | 4 (4.9%) | 0.1 |
| ALT*^c^* (U/L) | 32.5±18.4 | 29.6±19.4 | 0.4 |
| INR*^d^* | 1.1±0.1 | 1.0±0.1 | 0.07 |
| HBeAg | 1 (2.7%) | 0 (0%) | 0.3 |
| HBV DNA level |  |  | 0.3 |
| < 6 IU/mL | 6 (16.7%) | 21 (25.9%) |  |
| 6 – 1999 (IU/mL) | 30 (83.3%) | 60 (74.1%) |  |
| Duration of prophylaxis (mon.) | 9.0±3.4 | 8.3±3.8 | 0.3 |
| Follow-up (mon.) | 14.8±6.8 | 14.1±5.3 | 0.5 |

*^a^*Entecavir group included 5 head and neck cancer, 6 gynecologic cancers, and 1 genitourinary cancer. Lamivudine group included 15 head and neck cancers, 9 gynecologic cancers, and 10 genitourinary cancers.

*^b^*SCC: cytotoxic chemotherapy.

*^c^*ALT: alanine aminotransferase.

*^d^*INR: international normalized ratio.
